# Brain Morphometry in Infants Later Diagnosed With Autism is Related to Later Language Skills

**DOI:** 10.1002/hbm.70221

**Published:** 2025-05-09

**Authors:** Luke E. Moraglia, Bernadette Weigman, Hervé Abdi, Martin Styner, Sun Hyung Kim, Catherine A. Burrows, Mark D. Shen, Shruthi Ravi, Jason J. Wolff, Stephen R. Dager, Heather C. Hazlett, Juhi Pandey, Robert T. Schultz, Jessica B. Girault, Kelly N. Botteron, Natasha Marrus, Annette M. Estes, Tanya St. John, Guoyan Zheng, Joseph Piven, Meghan R. Swanson, the IBIS Network, J. Piven, H. C. Hazlett, C. Chappell, S. Dager, A. Estes, D. Shaw, K. Botteron, R. McKinstry, J. Constantino, J. Pruett, R. Schultz, J. Pandey, S. Paterson, L. Zwaigenbaum, J. Elison, J. Wolff, A. C. Evans, D. L. Collins, G. B. Pike, V. Fonov, P. Kostopoulos, S. Das, L. MacIntyre, G. Gerig, M. Styner, H. Gu

**Affiliations:** ^1^ Department of Psychology The University of Texas at Dallas Richardson Texas USA; ^2^ Department of Psychiatry University of North Carolina Chapel Hill North Carolina USA; ^3^ Department of Pediatrics University of Minnesota Minneapolis Minnesota USA; ^4^ Institute on Human Development and Disability University of Washington Seattle Washington USA; ^5^ Department of Educational Psychology University of Minnesota Minneapolis Minnesota USA; ^6^ Center for Autism Research The Children's Hospital of Philadelphia and University of Pennsylvania Philadelphia Pennsylvania USA; ^7^ Department of Psychiatry and Radiology Washington University St. Louis Missouri USA; ^8^ Institute of Medical Robotics School of Biomedical Engineering, Shanghai Jiao Tong University Shanghai China

**Keywords:** autism, cortical thickness, infants, language, surface area

## Abstract

Autism spectrum disorder (ASD) presents early in life with distinct social and language differences. This study explores the association between infant brain morphometry and language abilities using an infant‐sibling design. Participants included infants who had an older sibling with autism (high likelihood, HL) who were later diagnosed with autism (HL‐ASD; *n* = 31) and two non‐autistic control groups: HL‐Neg (HL infants not diagnosed with autism; *n* = 126) and LL‐Neg (typically developing infants who did not have an older sibling with autism; *n* = 77). Using a whole‐brain approach, we measured cortical thickness and surface area at 6 and 12 months and expressive and receptive language abilities at 24 months. Partial least squares correlation analyses were computed separately for each of the three groups. Results from the HL‐ASD group indicated negative associations between surface area in the left inferior frontal gyrus and 24‐month language abilities. Notably, regions outside the standard adult language network were also associated with language in the HL‐ASD group. Results in the HL‐ASD group highlight the distinct processing guiding development of surface area and cortical thickness; associations were mostly negative for surface area at 6 months but mostly positive for cortical thickness at the same time point. Results from this data‐driven study align with the theory of interactive specialization—a theory highlighting the dynamic nature of the infant brain—and advocate for a whole‐brain approach in investigating early brain‐behavior neurodevelopment in ASD.

## Graphical abstract

In this study, we found associations between cortical morphometry at 6 and 12 months of age and language abilities at 24 months of age in infants who either are or are not later diagnosed with autism. The patterns of brain regions associated with language were widespread throughout the brain and differed between the autistic and non‐autistic groups.

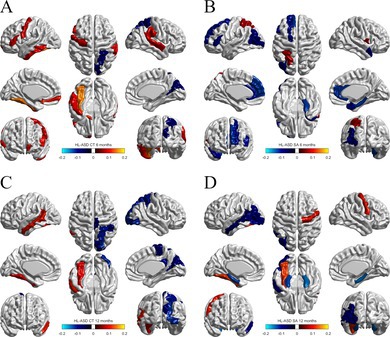

Autism spectrum disorder (ASD) is clinically characterized by differences in social communication and the presence of restrictive repetitive behaviors (DSM‐5; American Psychiatric Association 2013). Early social, behavioral, and cognitive markers of ASD are evident within the first 2 years of life (Ozonoff et al. 2010; Estes et al. 2015; Ravi et al. 2022) and can present as early as 9 months of age in infants later diagnosed with autism (Bradshaw et al. 2021). While clinical and associated features of autism are evident early in life, the average age of an autism diagnosis is about 4 years of age (Maenner et al. 2023).

Delays in early language are not part of the diagnostic criteria for ASD; however, for many infants who develop autism, delays in the comprehension and production of language occur early within the first 14 months of life (Mitchell et al. 2006; Hudry et al. 2014). Early challenges with language also persist for many autistic children: up to a third of all autistic children are either nonverbal or minimally verbal and will remain so throughout their lives (National Research Council 2001; Tager‐Flusberg and Kasari 2013; Rose et al. 2016).

Understanding the etiology of ASD‐associated language disruption during infancy could facilitate early identification efforts and inform the timing and targets of early language intervention. Importantly, socially informed efforts to assist language development in autistic individuals are supported and welcomed by many neurodiversity advocates (Kapp 2020). Despite the potential benefits of understanding the neurobiology of language in ASD during infancy, few studies have investigated the topic—a deficiency that results in a poor understanding of how the brain supports language in infants who develop autism.

One way to understand the neurobiology of early language is to identify associations between brain morphology and behavior. The morphometry of the cortex can be divided into two main measures: cortical thickness (CT, a measure of the thickness of cortical gray matter) and surface area (SA, a measure of the area covered by the cerebral cortex surface). In humans, there is rapid and dramatic growth of CT across the brain in the first 2 years of life, with the most growth occurring in the first 12 months after birth (Li et al. 2015; Lyall et al. 2015). Li et al. (2015) report that by 2 years of age, children have 97% of their adult CT values. Similarly, SA dramatically increases in the first 2 years of life, with children having 69% of their adult values by 2 years of age (Lyall et al. 2015).

Surface area and cortical thickness appear to be distinct phenotypes arising from unique processes. For example, in a neuroimaging study of neonates, bigger surface area was associated with being born male, higher birth weight, and later gestational age, whereas bigger cortical thickness values were associated with being born to a father with less educational attainment and being born to a white mother (vs. a Black mother; Jha et al. 2019). Interestingly—compared to typically developing (TD) control infants—infants who develop autism show larger surface area values and larger increases in SA from 6 to 12 months of age (Hazlett et al. 2017). In contrast, 9‐year‐old autistic children had smaller surface area values than typically developing controls. The typically developing controls in that study showed a decline in surface area across a 10‐year span, such that by 18 years of age, the ASD and TD surface area values were largely overlapping (Mensen et al. 2017). Taken together, SA development for autistic children differs from typically developing children at multiple points across the lifespan.

Despite several studies mapping SA and CT development in autistic and non‐autistic infants, fewer studies have specifically investigated language neurobiology through associations between brain and behavior. Language neurobiology of the adult brain has long been defined by three major structures: the inferior frontal gyrus (IFG), the superior temporal gyrus (STG), and the arcuate fasciculus (AF). The IFG (historically called Broca's area) is often associated with language production (Geschwind 1970; Tremblay and Dick 2016); the STG (historically called Wernicke's area) is often associated with language comprehension (Geschwind 1970; Tremblay and Dick 2016). The AF is a white matter tract with several segments directly and indirectly connecting the IFG and STG (Catani et al. 2005).

Both the IFG and STG are functionally activated in preverbal, 3‐month‐old infants when they listen to their native language—a pattern suggesting that the IFG also plays a role in language comprehension, at least during infancy (Dehaene‐Lambertz et al. 2002, 2006). While these studies provide evidence for a functional role of the IFG and STG in very young infants, there is also evidence that the structure of the STG is specialized for our species early in life as indicated, for example, by one study (Leroy et al. 2015) that compared the depth of the left and right superior temporal sulcus in humans and chimpanzees: Results provided evidence for lateralization that was present in very young human infants (*n* = 14), but not in adult chimpanzees. Research on adult participants has robustly reported that the superior temporal sulcus plays a key role in mapping sound to meaning (for a meta‐analysis see DeWitt and Rauschecker 2012), so if the region has a similar function during infancy, early specialization could aid in the rapid language development observed in the second year of life.

Many infant studies of the neurobiology of language restrict their investigations to the adult language network, often leaving unanswered the question of how other regions respond to language (Poeppel et al. 2012). But the infant brain is characterized by dynamic development and networks undergoing specialization, so studies focused on a limited number of regions may give an incomplete understanding of the neurobiology of language in infancy (Paterson et al. 2006). In fact, brain regions (such as the primary motor cortex and the posterior cingulate cortex) that are not typically associated with language abilities in adults have been implicated in expressive language abilities in 24‐month‐old typically developing infants (Girault et al. 2020). Additionally, theories expanding on the neuroscience of language suggest that many more aspects of the brain, such as multiple processing streams, are involved in language skills (Hickok and Poeppel 2000, 2004; Tremblay and Dick 2016)—a finding corroborated in adults (Saur et al. 2008).

To create a model of infant language neurobiology for autism, several key pieces of information need to be understood including (1) what regions are associated, at different times in development, with language skills in infants who develop autism, and (2) how brain‐behavior patterns of association differ in ASD and non‐ASD control groups. As a first step, the goal of the current study is to begin to address this gap by conducting a whole brain investigation of morphology associated with language. These associations are investigated using partial least squares correlation (PLSC)—an analytic approach that is ideally suited for studies investigating associations between many variables—separately in typically developing infants and in infants later diagnosed with ASD. We hypothesize that diffuse regions will be associated with language skills in both typically developing infants and in those who develop autism. Further, we hypothesize that typically developing infants will demonstrate primarily positive associations between cortical morphometry measures and later language skills.

## 1 Materials and Methods

### 1.1 Participants

This study includes data from a total of *N* = 234 infants comprising *n* = 157 infants with high familial likelihood (HL) for ASD and *n* = 77 with low familial likelihood (LL) for ASD. Data were collected as part of the Infant Brain Imaging Study (IBIS) across four clinical data sites: the University of North Carolina at Chapel Hill (UNC), the University of Washington in Seattle (SEA), the Children's Hospital of Philadelphia (PHI), and Washington University in St. Louis (STL). Parents provided written informed consent prior to participating in this study. The Institutional Review Boards at each site approved the study procedures.

The present study included all infants who had the following data available: (1) surface area and cortical thickness measurements from structural MRI brain scans at both ages 6 and 12 months, (2) language scores at 24 months, and (3) diagnostic assessments at 24 months. Infants were categorized into three groups based on clinical best‐estimate diagnoses. These diagnoses were made by licensed clinicians based upon DSM‐IV‐TR criteria after reviewing all available assessment data including the Autism Diagnostic Observation Schedule (ADOS; Lord et al. 2000; Lord et al. 2012), Autism Diagnostic Interview‐Revised (ADI‐R; Lord et al. 1994), Mullen Scales of Early Learning (MSEL; Mullen 1995), and the Vineland Adaptive Behavior Scales II (Sparrow et al. 2005). High familial likelihood ASD infants (HL‐ASD, *n* = 31) had an older sibling who met ASD criteria on the Social Communication Questionnaire (Rutter et al. 2003) and ADI‐R (Lord et al. 1994), with diagnosis confirmed by medical records, and they themselves received a clinical best‐estimate diagnosis of ASD at 24 months. High familial likelihood negative infants (HL‐Neg, *n* = 126) had an older sibling with ASD but did not receive a diagnosis of ASD at 24 months. Low familial likelihood negative infants (LL‐Neg, *n* = 77) scored 85 or above on the MSEL Early Learning Composite, had typically developing older siblings as determined by parent interview (Maxwell 1992), did not have first degree relatives with ASD, and did not receive a diagnosis of ASD at 24 months. Table 1 gives the demographics of the participants included in the final analyses.

**TABLE 1:** Descriptive data by group.

Variable	LL‐Neg (*n* = 77)	HL‐Neg (*n* = 126)	HL‐ASD (*n* = 31)	Test statistics
Age at scan [Mean (SD)]				
6‐mo visit	6.82 (0.68)	6.69 (0.70)	6.58 (0.72)	*F*(2, 231) = 1.477, *p* = 0.230
12‐mo visit	12.7 (0.78)	12.6 (0.61)	12.8 (0.68)	*F*(2, 231) = 1.643, *p* = 0.196
Age at MSEL [Mean (SD)]	24.7 (1.12)	24.7 (0.89)	24.6 (0.84)	*F*(2, 231) = 0.489, *p* = 0.614
MSEL *T*‐scores [Mean (SD)]				
Expressive language	55.2 (8.99)	49.3 (11.40)	38.0 (13.20)	*F*(2, 231) = 27.77, *p* < 10^−10^
Receptive language	59.3 (7.85)	53.1 (9.03)	33.4 (14.90)	*F*(2, 231) = 79.89, *p* < 10^−15^
Visual reception	59.5 (11.40)	54.0 (10.10)	42.4 (9.76)	*F*(2, 231) = 29.43, *p* < 10^−11^
Gross motor	55.3 (8.90)	50.9 (9.36)	39.9 (8.31)	*F*(2, 224) = 30.63, *p* < 10^−11^
Total SA 6 m [Mean (SD)]	61,524 (5307)	60,844 (5182)	63,258 (4230)	*F*(2, 231) = 2.817, *p* = 0.062
Total SA 12 m [Mean (SD)]	66,168 (4978)	65,991 (5006)	69,064 (4252)	*F*(2, 231) = 5.082, *p* = 0.007
ICV 6 m [Mean (SD)]	915,613 (98,058)	907,439 (87,991)	944,279 (76,661)	*F*(2, 231) = 2.083, *p* = 0.127
ICV 6 m [Mean (SD)]	1,074,904 (102,602)	1,070,503 (98,123)	1,122,223 (92,410)	*F*(2, 231) = 3.491, *p* = 0.032
ADOS [Mean (SD)]				
RRB‐CSS	2 (1.84)	2.83 (2.31)	6.43 (2.40)	*F*(2, 227) = 45.93, *p* < 10^−15^
SA‐CSS	1.64 (0.95)	1.67 (0.94)	6.17 (1.51)	*F*(2, 227) = 249.8, *p* < 10^−15^
Sex (% Male)	59.7%	54.8%	90.3%	χ^2^ = 13.329, *p* = 0.001
Mother's education level (%)				
High school/some college	10.4%	30.2%	38.7%	χ^2^ = 19.901, *p* = 0.0005
College degree/some graduate school	36.4%	40.5%	38.7%	
Graduate degree	53.2%	29.4%	22.6%	
Race (%)				
Black	3.9%	1.6%	0.0%	χ^2^ = 3.008, *p* = 0.0005
Asian	1.3%	0.8%	0.0%	
White	87.0%	88.0%	87.1%	
More than one race	7.8%	9.6%	12.9%	
Ethnicity Hispanic (%)	5.2%	7.1%	3.2%	χ^2^ = 0.806, *p* = 0.668
Site (%)				
PHI	16.9%	17.5%	25.8%	χ^2^ = 8.791, *p* = 0.186
SEA	24.7%	22.2%	32.3%	
UNC	37.7%	27.0%	16.1%	
STL	20.8%	33.3%	25.8%	

Exclusionary criteria included significant medical conditions known to affect brain development, sensory impairment, low birth weight (< 2200 g), prematurity (less than 36 weeks gestation), perinatal brain injury secondary to birth complications, exposure to specific medications or neurotoxins during gestation, non‐English speaking immediate family, contraindication for MRI, adoption, and first‐degree relative with psychosis, schizophrenia, or bipolar disorder. During data cleaning, additional participants were excluded: two low likelihood infants who met criteria for ASD and three low likelihood infants who had MSEL Early Learning Composite scores less than 85. These five infants were excluded from all analyses and are not included in the sample.

Two families had a pair of high likelihood siblings who both contributed MRI brain scans. One infant from each family was selected for inclusion in the current analyses based on the following criteria: (1) if one infant was HL‐ASD and the other was HL‐Neg, the HL‐ASD infant was kept in the analyses (due to a smaller sample size in the HL‐ASD group), (2) if both infants were HL‐Neg, the infant with more MRI data points was included, and (3) if both infants were HL‐Neg and had the same number of data points, a virtual coin toss was used to select the infant to include. From these criteria, one HL‐Neg infant was excluded from the analyses because they had an HL‐ASD sibling, and another was excluded based on a virtual coin toss. The previously reported sample sizes reflect one sibling from each sibling pair.

### 1.2 Procedures

Infants were scanned using MRI at 6 and 12 months of age and participated in a laboratory visit at 24 months of age. See Estes et al. (2015) for a full description of the assessment and diagnostic procedures.

### 1.3 Clinical Measures

Infant cognitive development was measured at 24 months using the Mullen Scales of Early Learning (MSEL, Mullen 1995)—a widely used assessment tool normed for children from birth to 68 months. The MSEL Early Learning Composite score measures overall cognitive development and comprises subscales evaluating expressive language, receptive language, fine motor skills, and visual reception. The key language measures for this study were the MSEL expressive language (EL) and receptive language (RL) *T*‐scores. Some HL‐ASD infants were at floor for the EL and RL *T*‐scores, but analyses using MSEL age‐equivalent (AE) scores without participants at floor showed very similar results for all groups (Tables S2, S3, S4, and S5).

The ADOS is a semi‐structured observational play assessment of social interaction, communication, and repetitive behaviors (Lord et al. 2000, 2012). ADOS‐G was administered for assessments conducted before 2012, and the ADOS‐2 was used for assessments after that date. Module 1 or 2 was administered for all participants at 24 months, and conventional scoring algorithms were applied (Gotham et al. 2007). To provide information on participant characteristics, Table 1 includes means, by group, for the ADOS Social Affect calibrated severity score (ADOS SA‐CSS) and the ADOS Restricted and Repetitive Behavior calibrated severity score (ADOS RRB‐CSS; Hus et al. 2014).

### 1.4 MRI Acquisition and Processing

Pediatric imaging was completed during natural sleep at each clinical site using identical 3‐T Siemens TIM Trio scanners. T1 and T2‐weighted scans (1 mm^3^ voxels) were acquired (a full description of the MRI acquisition and image preprocessing can be found in the supplemental material; see also Hazlett et al. 2017, for a description of the acquisition and processing procedures).

Several quality control procedures were employed to assess scanner stability and reliability across sites, time, and procedures. Geometric phantoms were scanned monthly and human phantoms (two adult participants) were scanned annually to monitor scanner stability at each site across the study period. Full details on the stability procedures for IBIS and scanner quality control checks are described by Hazlett et al. (2012). Because of these a priori quality control procedures, it was unnecessary to use additional harmonization techniques (such as ComBat; Johnson et al. 2007; Fortin et al. 2018) intended to account for different scanner manufacturers or magnet strengths. As an added precaution, however, site was used as a covariate in the statistical analyses.

### 1.5 Regional Measures

Cortical surfaces were generated using an adapted version of the CIVET processing pipeline (Kim et al. 2005). The subsequent white matter (WM) and gray matter (GM) surfaces were characterized by the presence of 327,680 high‐definition triangular mesh configurations (comprising 163,842 vertices) in each cerebral hemisphere. These surfaces facilitated the procurement of inherent cortical thickness and surface area metrics.

Correspondence of the cortical surface was ascertained through a process of spherical registration, conformed to an averaged surface template. This process implements sphere‐to‐sphere warping by aligning the apices of gyri (Robbins 2004)—a procedure achieved by computing the mean of surfaces derived from all study participants.

For the purposes of quality control, both WM and GM surfaces were subjected to visual assessment slice‐by‐slice by overlapping the T1/T2 scans of each subject with the WM‐GM and GM‐cerebrospinal fluid boundary surfaces. Cortical surfaces were assigned a quality score (ranging from 0 to 3) based on the following criteria: (0) The surface itself was not created, (1) the WM surface and the GM surface were intersected, or the GM surface was under‐ or over‐estimated by more than 3 voxels (especially in the temporal tip and inferior frontal areas), or the WM surface was disconnected, (2) minor issues, such as each boundary under‐ or over‐estimated within 1–2 voxels, and (3) no errors. Surfaces assigned a 0 or 1 were excluded from analyses. At 6 months, 57 out of 466 total scans were excluded, and at 12 months, 14 out of 458 total scans were excluded. At both time points, there was no difference in the exclusion rate between participants who went on to have an ASD diagnosis and those who did not receive an ASD diagnosis [exclusion rate 6 months: ASD: 3.7%, Non‐ASD: 9.6%, χ^2^(1) = 1.99, *p* = 0.159; 12 months: ASD: 4.0%, Non‐ASD: 3.0%, χ^2^(1) = 0.16, *p* = 0.694].

In the final phase, 78 regional cerebral measurements were determined by the summation of the area and the computation of the mean thickness within each designated region. This was achieved utilizing an age‐adjusted Automated Anatomical Labeling (AAL) atlas (Tzourio‐Mazoyer et al. 2002).

### 1.6 Partial Least Squares Correlation (PLSC) Analysis

Relationships between regional brain morphometry (i.e., SA and CT) and language ability (i.e., MSEL scores) were analyzed using PLSC, a multivariate method frequently used in neuroimaging since its introduction by McIntosh et al. (1996). PLSC is well‐suited for examining relationships between two tables of quantitative variables and can be thought of as a generalization of correlation to the case of two data tables. In‐depth overviews of PLSC are available (McIntosh and Lobaugh 2004; Krishnan et al. 2011; Abdi and Williams 2013), and here we only give a brief description of the key points.

PLSC analyzes the relationships between two data tables (collected on the same set of observations): here a table of brain measurements (in this case, SA and CT measurements) and a table of behavioral measurements (in this case, MSEL scores). From each table, a new variable—called a latent variable—is computed such that the latent variable from the brain table and the latent variable from the behavioral table have maximal covariance and therefore best capture the similarities between the two tables. Each latent variable is computed as a linear combination of either the brain or behavior variables, and the weight—called a loading—in the linear combination given to a variable quantifies the importance of this variable for that latent variable, with larger loadings indicating greater importance. When two variables have loadings with the same sign (i.e., both positive or both negative), then there is a positive relationship between these variables captured by the latent variable, whereas loadings with opposite signs indicate negative relationships.

PLSC can create several pairs of latent variables, which are obtained in order of decreasing importance based on their covariance. Mathematically, the latent variables are obtained from the singular value decomposition (SVD; Greenacre 1984; Abdi 2007) of the correlation matrix between the two data tables. The SVD provides left singular vectors (which are the loadings for the behavior latent variables), right singular vectors (which are loadings for the brain latent variables), and singular values (which are the covariances between the pairs of latent variables).

To identify the pairs of latent variables that explained a significant covariance between the brain and behavioral tables, we ran a permutation test on the eigenvalue proportion (EVP) of each pair of latent variables, a test that has recently shown promise for its low false positive rate and its power to correctly detect latent variables that capture true relationships (Moraglia 2024, pending publication). Permutation tests have frequently been used with PLSC, most often by testing the singular values (McIntosh and Lobaugh 2004; Krishnan et al. 2011), but this test has recently been challenged on both its false positive rate and its power (McIntosh 2021; Moraglia 2024, pending publication). The EVP of a pair of latent variables is computed as the eigenvalue (squared singular value) of the pair divided by the sum of all the eigenvalues. The permutation test works by permuting the values of each variable of both data tables, so that the relationships between the two tables are broken. These permuted data tables are analyzed with PLSC, and the EVP for each pair of latent variables is computed and stored. This permutation process repeats many times (here 1000 times) to create a null distribution of EVP values for each pair of latent variables. These null distributions are then used for null hypothesis testing, and pairs of latent variables are considered significant if the resulting *p* value was less than 0.01.

To assess the significance of the variable loadings, bootstrap ratios were computed using a bootstrap resampling procedure (McIntosh and Lobaugh 2004; Krishnan et al. 2011; Abdi and Williams 2013). This procedure (1) creates new pairs (e.g., here 1000) of matched data tables (with the same sample size as the original data) by sampling with replacement the original observations, (2) analyzes (with PLSC) each pair of tables, and (3) stores the variable loadings. Then, for each variable, the bootstrap procedure computes the mean and standard deviation of its (bootstrapped) distribution and its bootstrap ratio by dividing its mean by its standard deviation. This ratio is similar to a Student's *t* criterion, so it quantifies the stability of the loadings: a ratio with a large magnitude signals an important variable. For example, bootstrap ratios outside the interval [−2, 2] are considered significant, because these values approximately matches a two‐tailed probability of *p* < 0.05 for a Student's *t* criterion (Abdi and Williams 2013). Additionally, 95% confidence intervals of the loadings were computed from the bootstrap iterations.

We ran a separate PLSC analysis for each diagnostic group: LL‐Neg, HL‐Neg, and HL‐ASD. We chose to run separate analyses for each group because we were interested in the relationships between morphometry and language ability within each group, rather than the relationships across all individuals. Diagnostic group was highly related to language ability (Table 1), so relationships between morphometry and language across all individuals would be mostly driven by differences in diagnosis. Instead, we report statistical relationships between morphometry and language within group.

The variables in each table were the same for each of the three analyses. The brain table contained the measurements of cortical thickness and surface area at 6 and 12 months for 78 regions in the AAL atlas, for a total of 312 measurements, and the behavior table contained the MSEL RL and EL *T*‐scores.

Prior to PLSC, we corrected brain and language variables by regressing out the linear effects of several covariates using linear regression. For all variables, we corrected for sex, mother's education level, data collection site, and age at data collection. Additionally, surface area measurements were corrected for total surface area, and cortical thickness measurements were corrected with the cube root of the intracranial volume. The resulting residuals of the linear regression analyses were used as the input variables for the PLSC analyses. All linear regression analyses were done within each diagnostic group.

All analyses were conducted using R 4.2.1 (R Core Team 2022). The PLSC analyses were conducted using the TExPosition package (Beaton et al. 2014), the permutation tests using the componentts package (Moraglia 2024, pending publication), and bootstrap resampling using the data4PCCAR package (Abdi and Beaton 2025, pending publication). Figures of significant brain regions were created using the SurfStat toolbox (Worsley et al. 2009) in MATLAB 9.9.0.1524771 (The MathWorks Inc. 2020).

## 2 Results

### 2.1 PLSC LL‐Neg Group

For the LL‐Neg group, the first pair of latent variables (LV1) was significant based on the permutation test, *p* = 0.006, and had a covariance equal to 2.46 corresponding to an EVP of 67.47%. Loadings for expressive and receptive language were both positive for LV1 (EL: 0.733 and RL: 0.680)—a pattern indicating a positive relationship between the language variables, and both loadings were significant based on their bootstrap ratios (EL: 4.923 and RL: 3.678). These positive loadings for the language variables indicate that the first language latent variable is akin to an optimal composite score for the two language measures (the same interpretation applies to the analyses on the other diagnostic groups).

**FIGURE 1:**
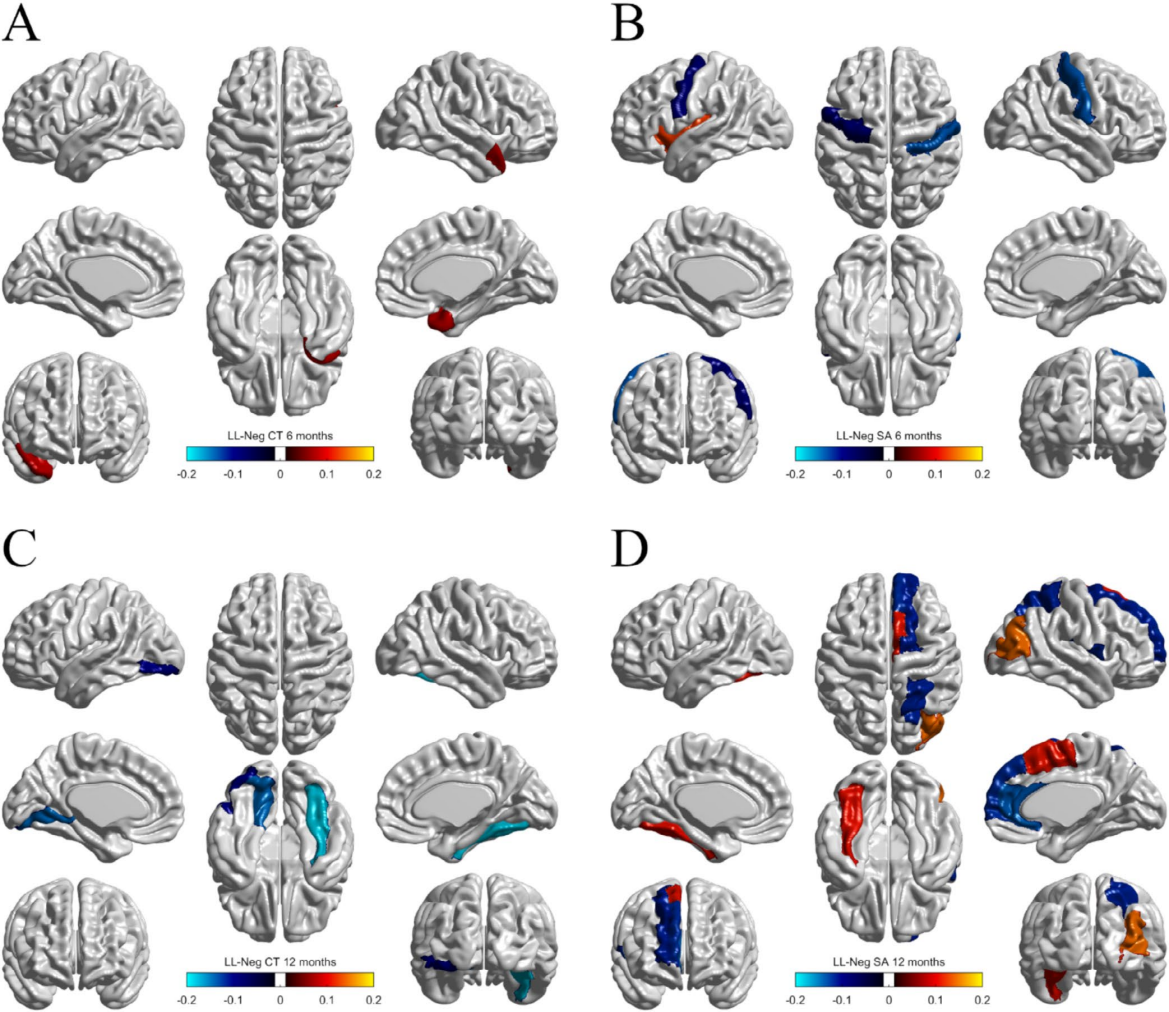
LL‐Neg brain regions associated with language abilities, colored by their loadings on LV1. Blue regions were negatively associated, while red/orange regions were positively associated. (A) 6‐month CT. The right temporal pole of the superior temporal gyrus was positively associated. (B) 6‐month SA. Two regions were negatively associated: The left precentral gyrus and right postcentral gyrus. The left insula was positively associated. (C) 12‐month CT. Three regions were negatively associated: The left lingual gyrus, left inferior occipital gyrus, and right fusiform gyrus. (D) 12‐month SA. Seven of eight regions were right lateralized. Five regions were negatively associated: The right dorsolateral and medial superior frontal gyri, right Rolandic operculum, right anterior cingulate and paracingulate gyri, and right superior parietal gyrus. Three regions were positively associated: The right supplementary motor area, right middle occipital gyrus, and left fusiform gyrus.

For brain morphometry, loadings and bootstrap ratios identified eight regional surface area variables at 12 months that were associated with language abilities (Figure 1D), seven of which were right lateralized. Additionally, three 6‐month surface area variables, one 6‐month cortical thickness variable, and three 12‐month cortical thickness variables were associated with language on LV1 (Figure 1). None of the regions were associated with language at both time points. The full list of significant brain regions is given in Table 2.

**TABLE 2:** LL‐Neg brain morphometry LV1 loadings and bootstrap ratios.

Region	Morphometry	Loading 6 m	Bootstrap ratio 6 m	Loading 12 m	Bootstrap ratio 12 m
Left precentral gyrus	Surface area	**−0.083 [−0.165, −0.009]**	**−2.110**	−0.047 [−0.137, 0.063]	−0.907
Left insula		**0.123 [0.028, 0.226]**	**2.367**	0.102 [−0.014, 0.238]	1.578
Right postcentral gyrus		**−0.135 [−0.258, −0.028]**	**−2.315**	−0.078 [−0.183, 0.020]	−1.475
Right temporal pole: superior temporal gyrus	Cortical thickness	**0.080 [0.007, 0.157]**	**2.042**	0.058 [−0.062, 0.173]	0.964
Right superior frontal gyrus, dorsolateral	Surface area	−0.037 [−0.134, 0.054]	−0.809	**−0.112 [−0.228, −0.014]**	**−2.082**
Right rolandic operculum		−0.065 [−0.174, 0.034]	−1.204	**−0.116 [−0.220, −0.021]**	**−2.280**
Right supplementary motor area		0.107 [0.008, 0.210]	1.955	**0.109 [0.027, 0.196]**	**2.453**
Right superior frontal gyrus, medial		−0.016 [−0.098, 0.079]	−0.361	**−0.119 [−0.227, −0.015]**	**−2.165**
Right anterior cingulate and paracingulate gyri		−0.075 [−0.182, 0.033]	−1.371	**−0.136 [−0.257, −0.030]**	**−2.383**
Right middle occipital gyrus		0.085 [−0.018, 0.179]	1.661	**0.142 [0.038, 0.243]**	**2.683**
Left fusiform gyrus		0.006 [−0.097, 0.112]	0.130	**0.106 [0.015, 0.205]**	**2.147**
Right superior parietal gyrus		−0.063 [−0.182, 0.061]	−1.059	**−0.115 [−0.235, 0.002]**	**−2.001**
Left lingual gyrus	Cortical thickness	0.011 [−0.091, 0.106]	0.251	**−0.141 [−0.277, −0.021]**	**−2.197**
Left inferior occipital gyrus		−0.055 [−0.163, 0.053]	−1.040	**−0.099 [−0.195, −0.000]**	**−2.004**
Right fusiform gyrus		−0.022 [−0.120, 0.068]	−0.486	**−0.180 [−0.347, −0.058]**	**−2.433**

*Note:* Bold indicates bootstrap ratios outside the interval [–2 2], which is considered significant.

### 2.2 PLSC HL‐Neg Group

For the HL‐Neg group, LV1 was significant based on the permutation test, *p* = 0.001, and had a covariance equal to 2.20 corresponding to an EVP of 83.57%. Loadings for expressive and receptive language were both positive for LV1 (EL: 0.793 and RL: 0.609)—a pattern indicating a positive relationship between the language variables—and both loadings were significant based on their bootstrap ratios (EL: 5.649 and RL: 4.012).

**FIGURE 2:**
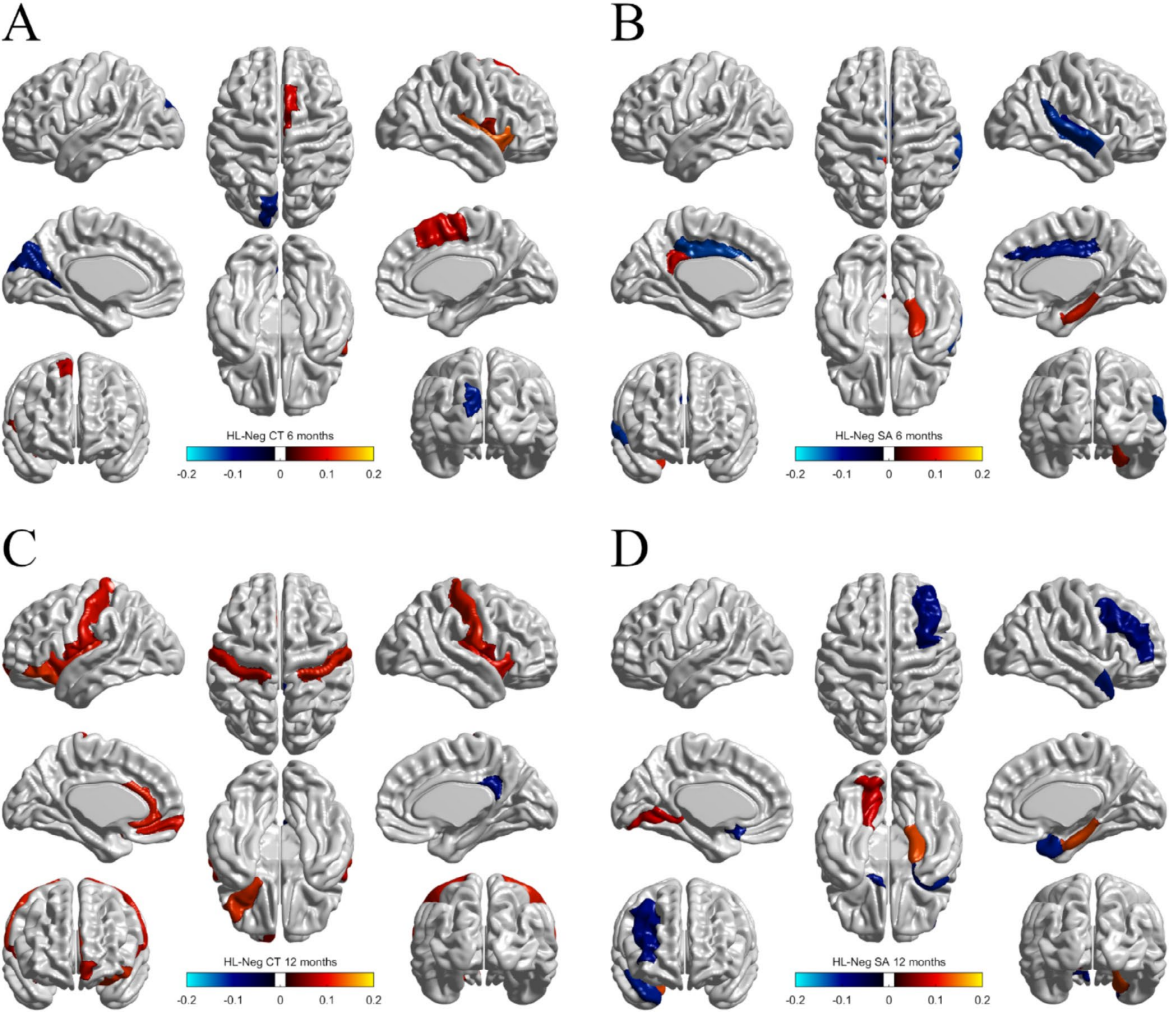
HL‐Neg brain regions associated with language abilities, colored by their loadings on LV1. Blue regions were negatively associated, while red/orange regions were positively associated. (A) 6‐month CT. Three regions were positively associated: The right Rolandic operculum, right supplementary motor area, and right insula. The left cuneus was negatively associated. (B) 6‐month SA. Four regions were negatively associated: The left and right median cingulate and paracingulate gyri, right Heschl gyrus, and right superior temporal gyrus. Two regions were positively associated: The left posterior cingulate gyrus and right parahippocampal gyrus. (C) 12‐month CT. Nine regions were positively associated: The left inferior frontal gyrus (orbital part), left and right Rolandic operculum, left medial orbital superior frontal gyrus, left and right insula, left anterior cingulate and paracingulate gyri, and left and right postcentral gyri. The right posterior cingulate gyrus was negatively associated. (D) 12‐month SA. Three regions were negatively associated: The right middle frontal gyrus, left olfactory cortex, and right temporal pole of the superior temporal gyrus. Two regions were positively associated: The right parahippocampal gyrus and left lingual gyrus.

For brain morphometry, loadings and bootstrap ratios identified several regions associated with language abilities for surface area and cortical thickness variables at both time points (Figure 2). Of these regions, 10 were 12‐month cortical thickness variables, of which nine were positively associated with language (Figure 2C). The CT of the right Rolandic operculum and the right insula, as well as the SA of the right parahippocampal gyrus, were associated with language at both time points. The CT of the left inferior frontal gyrus, orbital part, at 12 months was positively associated with language; this is the only significant region that is part of the classic model of language neurobiology. The full list of significant brain regions is available in Table 3.

**TABLE 3:** HL–Neg brain morphometry LV1 loadings and bootstrap ratios.

Region	Morphometry	Loading 6 m	Bootstrap ratio 6 m	Loading 12 m	Bootstrap ratio 12 m
Left median cingulate and paracingulate gyri	Surface area	**−0.127 [−0.229, −0.038]**	**−2.473**	−0.095 [−0.207, −0.003]	−1.776
Right median cingulate and paracingulate gyri		**−0.110 [−0.211, −0.006]**	**−2.033**	−0.047 [−0.138, 0.045]	−1.012
Left posterior cingulate gyrus		**0.103 [0.012, 0.196]**	**2.216**	0.084 [−0.026, 0.173]	1.599
Right parahippocampal gyrus		**0.113 [0.014, 0.212]**	**2.227**	**0.130 [0.043, 0.227]**	**2.818**
Right Heschl gyrus		**−0.100 [−0.195, −0.016]**	**−2.311**	−0.030 [−0.125, 0.059]	−0.641
Right superior temporal gyrus		**−0.120 [−0.219, −0.025]**	**−2.424**	−0.095 [−0.200, −0.001]	−1.896
Right Rolandic operculum	Cortical thickness	**0.113 [0.012, 0.215]**	**2.205**	**0.097 [0.011, 0.189]**	**2.025**
Right supplementary motor area		**0.102 [0.013, 0.198]**	**2.160**	0.042 [−0.053, 0.140]	0.853
Right insula		**0.135 [0.038, 0.246]**	**2.573**	**0.105 [0.012, 0.204]**	**2.103**
Left cuneus		**−0.112 [−0.201, −0.027]**	**−2.652**	−0.054 [−0.145, 0.027]	−1.236
Right middle frontal gyrus	Surface area	−0.021 [−0.102, 0.058]	−0.548	**−0.105 [−0.197, −0.015]**	**−2.212**
Left olfactory cortex		−0.038 [−0.125, 0.047]	−0.912	**−0.107 [−0.203, −0.017]**	**−2.283**
Left lingual gyrus		0.086 [−0.008, 0.177]	1.815	**0.098** **[0.004, 0.182]**	**2.182**
Right temporal pole: superior temporal gyrus		−0.044 [−0.141, 0.056]	−0.884	**−0.112 [−0.206, −0.017]**	**−2.254**
Left inferior frontal gyrus, orbital part	Cortical thickness	0.031 [−0.076, 0.139]	0.590	**0.120 [0.029, 0.225]**	**2.490**
Left Rolandic operculum		0.061 [−0.041, 0.171]	1.165	**0.106 [0.009, 0.212]**	**2.013**
Left superior frontal gyrus, medial orbital		−0.054 [−0.166, 0.054]	−0.970	**0.108 [0.016, 0.198]**	**2.320**
Left insula		0.134 [0.001, 0.282]	1.862	**0.110 [0.023, 0.202]**	**2.353**
Left anterior cingulate and paracingulate gyri		−0.040 [−0.162, 0.084]	−0.635	**0.111 [0.016, 0.210]**	**2.158**
Right posterior cingulate gyrus		−0.024 [−0.110, 0.055]	−0.603	**−0.105 [−0.194, −0.021]**	**−2.431**
Left postcentral gyrus		0.069 [−0.021, 0.157]	1.471	**0.106 [0.009, 0.212]**	**2.172**
Right postcentral gyrus		0.063 [−0.023, 0.156]	1.361	**0.112 [0.029, 0.208]**	**2.423**

*Note:* Bold indicates bootstrap ratios outside the interval [–2 2], which is considered significant.

### 2.3 PLSC HL‐ASD Group

For the HL‐ASD group, LV1 was significant based on the permutation test, *p* = 0.001, and had a covariance equal to 4.99 corresponding to an EVP of 87.43%. Loadings for expressive and receptive language were both positive for LV1 (EL: 0.700 and RL: 0.714), indicating a positive relationship between the language variables, and both loadings were significant based on their bootstrap ratios (EL: 3.391 and RL: 5.027).

**FIGURE 3:**
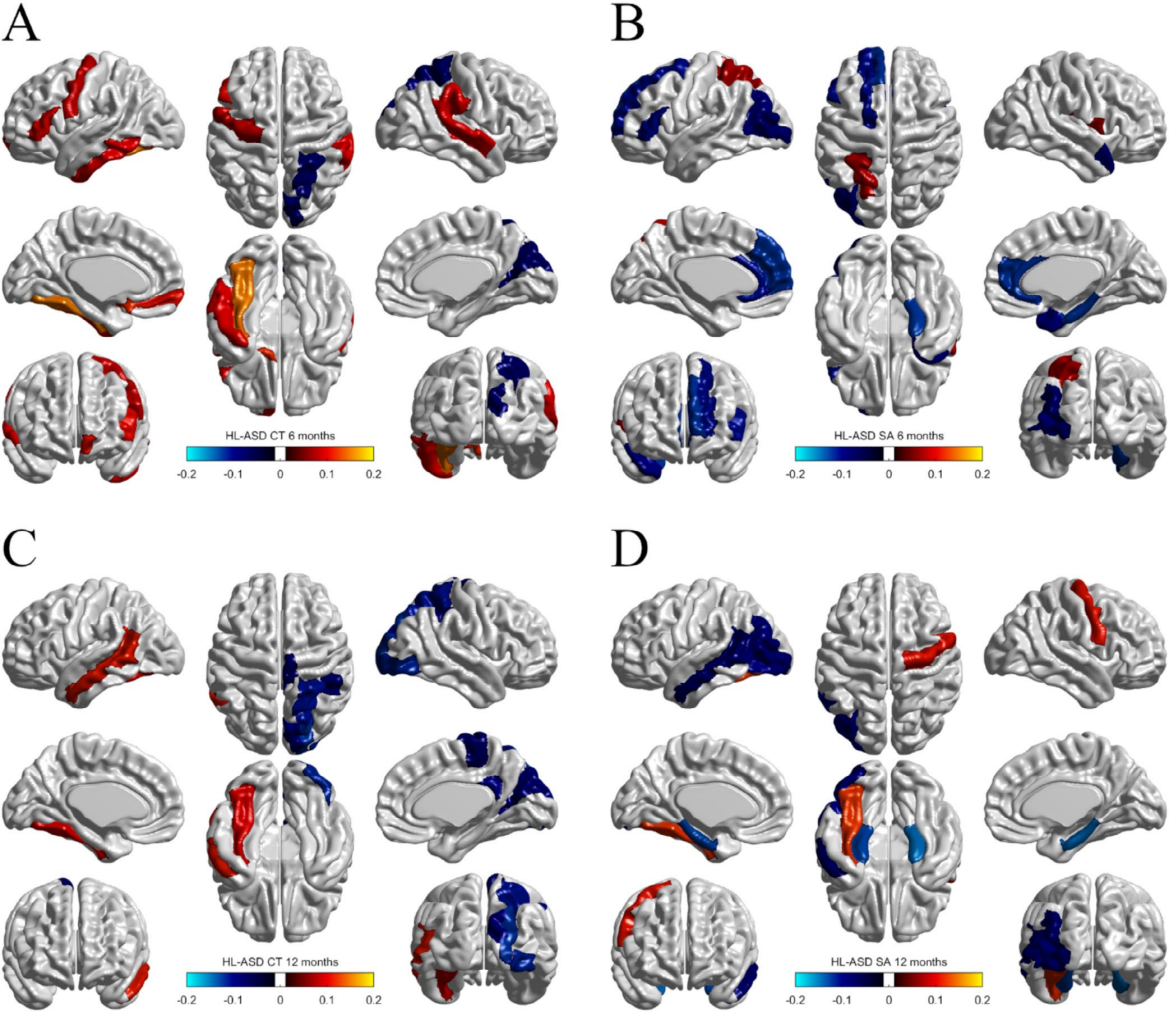
HL‐ASD brain regions associated with language abilities, colored by their loadings on LV1. Blue regions were negatively associated, while red/orange regions were positively associated. (A) 6‐month CT. Eight regions were positively associated: The left precentral gyrus, left inferior frontal gyrus (triangular part), left olfactory cortex, left medial orbital superior frontal gyrus, left fusiform gyrus, right supramarginal gyrus, right superior temporal gyrus, and left inferior temporal gyrus. Two regions were negatively associated: The right cuneus and right superior parietal gyrus. (B) 6‐month SA. Eight regions were negatively associated: The left dorsolateral and medial superior frontal gyri, left inferior frontal gyrus (triangular part), left and right anterior cingulate and paracingulate gyri, right parahippocampal gyrus, left middle occipital gyrus, and right temporal pole of the superior temporal gyrus. Two regions were positively associated: The right Rolandic operculum and left superior parietal gyrus. (C) 12‐month CT. Seven regions were negatively associated: The right posterior cingulate gyrus, right cuneus, right superior occipital gyrus, right inferior occipital gyrus, right superior parietal gyrus, right inferior parietal (but supramarginal and angular) gyri, and right paracentral lobule. Two regions were positively associated: The left fusiform gyrus and left middle temporal gyrus. (D) 12‐month SA. Five regions were negatively associated: The left and right parahippocampal gyri, left middle occipital gyrus, left inferior occipital gyrus, and left middle temporal gyrus. Two regions were positively associated: The right precentral gyrus and left fusiform gyrus.

For brain morphometry, loadings and bootstrap ratios identified surface area and cortical thickness variables at both time points that were both positively and negatively related to language (Figure 3). The 6‐month SA, 12‐month SA, and 12‐month CT regions were mostly negatively related to language, whereas 6‐month CT regions were mostly positively related to language. The CT of the right cuneus, left fusiform gyrus, and right superior parietal gyrus, as well as the SA of the right parahippocampal gyrus and left middle occipital gyrus were associated with language at both time points. The CT and SA of the left inferior frontal gyrus, triangular part, at 6 months were associated with language; this is the only significant region that is part of the classic model of language neurobiology. The full list of significant brain regions is available in Table 4.

**TABLE 4:** HL‐ASD brain morphometry LV1 loadings and bootstrap ratios.

Region	Morphometry	Loading 6 m	Bootstrap ratio 6 m	Loading 12 m	Bootstrap ratio 12 m
Left superior frontal gyrus, dorsolateral	Surface area	**−0.104 [−0.205, −0.019]**	**−2.208**	−0.018 [−0.107, 0.070]	−0.389
Left inferior frontal gyrus, triangular part		**−0.105 [−0.190, −0.034]**	**−2.585**	0.014 [−0.065, 0.089]	0.341
Right Rolandic operculum		**0.082 [0.009, 0.169]**	**2.021**	0.012 [−0.060, 0.086]	0.321
Left superior frontal gyrus, medial		**−0.121 [−0.196, −0.053]**	**−3.341**	−0.005 [−0.097, 0.079]	−0.116
Left anterior cingulate and paracingulate gyri		**−0.105 [−0.185, −0.035]**	**−2.724**	−0.050 [−0.149, 0.038]	−1.051
Right anterior cingulate and paracingulate gyri		**−0.120 [−0.230, −0.022]**	**−2.275**	−0.011 [−0.107, 0.080]	−0.252
Right parahippocampal gyrus		**−0.122 [−0.223, −0.034]**	**−2.490**	**−0.143 [−0.277, −0.040]**	**−2.405**
Left middle occipital gyrus		**−0.087 [−0.172, −0.005]**	**−2.032**	**−0.089 [−0.168, −0.003]**	**−2.130**
Left superior parietal gyrus		**0.077 [0.016, 0.143]**	**2.378**	0.055 [−0.019, 0.137]	1.387
Right temporal pole: superior temporal gyrus		**−0.096 [−0.181, −0.017]**	**−2.420**	−0.033 [−0.105, 0.048]	−0.785
Left precentral gyrus	Cortical thickness	**0.086 [0.017, 0.152]**	**2.427**	0.003 [−0.075, 0.082]	0.041
Left inferior frontal gyrus, triangular part		**0.104 [0.012, 0.200]**	**2.106**	0.004 [−0.084, 0.092]	0.078
Left olfactory cortex		**0.117 [0.019, 0.202]**	**2.525**	−0.073 [−0.187, 0.019]	−1.483
Left superior frontal gyrus, medial orbital		**0.107 [0.026, 0.192]**	**2.443**	0.012 [−0.072, 0.098]	0.254
Right cuneus		**−0.080 [−0.149, −0.003]**	**−2.130**	**−0.091 [−0.165, −0.017]**	**−2.304**
Left fusiform gyrus		**0.148 [0.044, 0.253]**	**2.844**	**0.094 [0.022, 0.165]**	**2.431**
Right superior parietal gyrus		**−0.080 [−0.148, −0.015]**	**−2.277**	**−0.108 [−0.198, −0.023]**	**−2.300**
Right supramarginal gyrus		**0.100 [0.017, 0.188]**	**2.320**	−0.050 [−0.124, 0.027]	−1.263
Right superior temporal gyrus		**0.098 [0.026, 0.180]**	**2.420**	−0.052 [−0.123, 0.029]	−1.361
Left inferior temporal gyrus		**0.099 [0.004, 0.190]**	**2.112**	0.079 [−0.032, 0.193]	1.343
Right precentral gyrus	Surface area	0.097 [0.010, 0.203]	1.968	**0.108 [0.034, 0.183]**	**2.770**
Left parahippocampal gyrus		−0.010 [−0.107, 0.092]	−0.183	**−0.128** **[−0.249, −0.021]**	**−2.269**
Left inferior occipital gyrus		−0.015 [−0.107, 0.083]	−0.302	**−0.100 [−0.196, −0.010]**	**−2.053**
Left fusiform gyrus		0.064 [−0.019, 0.140]	1.569	**0.121 [0.028, 0.219]**	**2.569**
Left middle temporal gyrus		−0.013 [−0.089, 0.067]	−0.360	**−0.084 [−0.155, −0.016]**	**−2.329**
Right posterior cingulate gyrus	Cortical thickness	0.040 [−0.052, 0.144]	0.820	**−0.102 [−0.173, −0.037]**	**−2.851**
Right superior occipital gyrus		0.021 [−0.056, 0.098]	0.561	**−0.120 [−0.210, −0.032]**	**−2.639**
Right inferior occipital gyrus		0.018 [−0.063, 0.104]	0.440	**−0.115 [−0.193, −0.035]**	**−2.789**
Right inferior parietal, but supramarginal and angular gyri		0.052 [−0.022, 0.128]	1.358	**−0.089 [−0.172, −0.008]**	**−2.094**
Right paracentral lobule		0.039 [−0.039, 0.121]	0.931	**−0.079 [−0.158, −0.009]**	**−2.061**
Left middle temporal gyrus		0.044 [−0.034, 0.131]	1.007	**0.110 [0.032, 0.204]**	**2.471**

*Note:* Bold indicates bootstrap ratios outside the interval [–2 2], which is considered significant.

## 3 Discussion

The objective of this study was to move the field a step closer to a neurobiological model of language for infants who develop autism. Towards this end, we conducted a data‐driven study investigating the associations between 6 and 12 month whole‐brain regional CT and SA and 24 month receptive and expressive language skills in infants who developed autism (HL‐ASD). To provide qualitative information, we also tested these associations in two control groups using separate models for each group (HL‐Neg and LL‐Neg).

A visual depiction of the significant regions for each of the three groups can be found in Figure S1. Importantly, results indicated widespread brain regions associated with language abilities. These regions were outside of the putative language networks most often identified in adults. Brain regions often varied across time points and between CT and SA. Additionally, the direction of association with language abilities, positive or negative, varied across measures.

Previous studies of both infants and adults have reported regional and developmental differences in the associations between cortical morphometry and cognitive skills. In a large cohort of healthy infants, thicker cortices at 1 and 2 years of age and larger brain surface areas at birth, 1, and 2 years of age were associated with increased cognitive ability—including language skills—at 2 years (Girault et al. 2020). This same pattern of positive associations has been reported for older children and adolescents (Burgaleta et al. 2014; Schnack et al. 2015). However, longitudinal studies have reported dynamic patterns of association between cortical thickness and intelligence (Shaw et al. 2006; Menary et al. 2013). Shaw et al. (2006) reported that for young children (i.e., 3–8 years of age) cortical thickness was negatively associated with intelligence, but for older children and adults there was a positive association between cortical thickness and intelligence. Regional differences in patterns of association for the young children were also reported, with stronger negative associations in frontal and temporal regions (Shaw et al. 2006).

Understanding the morphological substrates of ASD in children and adults has been the focus of a growing body of literature. Arutiunian et al. (2023) observed an increase in cortical thickness in language‐related brain regions in association with better language abilities in autistic children at school age. Balardin et al. (2015) reported thicker dorsal and temporal cortices and thinner lateral orbito‐frontal and parieto‐occipital cortices in association with higher verbal IQ scores in autistic adults. While the current study did find regional positive associations between brain morphometry and language skills in HL‐ASD infants, there were many regions that were negatively associated with language skills in infants who went on to have autism—a configuration suggesting that the anatomical specificity of the neural correlates of language is variable.

The superior temporal gyrus (STG) is a region often associated with language skills and hence warrants closer examination. The current study found that the STG was associated with language abilities in all three groups, but the nature of this association differed by time point, morphometry measure, and direction of effect (Table S1). At 6 months of age, the surface area of the right STG was negatively associated with language skills in HL‐ASD and HL‐Neg infants. This negative association between right STG SA and language was also found at 12 months of age in the HL‐Neg infants. However, right STG CT at 6 months of age was *positively* associated with language skills in LL‐Neg and HL‐ASD infants. These results are consistent with a large body of work showing that the superior temporal gyrus plays a role in receptive language abilities (for review, see Hickock and Poeppel 2007). At least one study has found that the pattern of association between STG and language skills was moderated by group. Bigler et al. (2007) reported that, at the group level, autistic children showed no association between STG volume and language abilities whereas individuals without ASD showed a significant association between STG volume and language abilities.

Overall, our results suggest that both typically developing infants and infants who go on to have ASD do not have highly specialized language structures. Definitions of brain specialization vary, but common elements include the relationship between brain region and mechanism is “innate, domain‐specific, and isolated from other brain systems” (Barrett 2012). Our results align more with the theory of interactive specialization, which provides a theoretical framework to understanding how the brain changes as humans mature. The theory states that the brain specializes through both time and experience, and, as a result, structure–function associations also change (Johnson 2011). Despite scientific support for the theory of interactive specialization (Battista et al. 2018), little developmental language research has been conducted using a structural global brain exploration approach. However, functionally, the idea that the infant brain differs from the adult brain, especially in high‐order networks, has been supported by a few notable infant studies (Redcay et al. 2008; Gao et al. 2015). For example, typically developing 1‐ and 2‐year‐old toddlers had widespread functional activation of brain regions outside of the Perisylvian network when listening to forward speech. This pattern was markedly different in 3‐year‐old children who showed more adult‐like functional activation when listening to forward speech (Redcay et al. 2008). Future efforts should expand upon the current work by investigating longitudinal patterns of brain morphometry and associations with language skills.

### 3.1 Alternative Approaches

When compared to typically developing infants, HL‐Neg infants are 3 to 4 times more likely to display delays in language skills (Marrus et al. 2018). Additionally, we previously reported that the sub‐cortical brain and behavior of HL‐Neg infants with a history of language delay are phenotypically different from HL‐ASD infants (Swanson et al. 2017). It is possible, even likely, that results would differ if the HL‐Neg group were to be further separated into subgroups based on possible language delay. Although beyond the scope of this study, future research should consider addressing this question. Future studies could also use the results herein to reduce the feature space for more targeted, hypothesis‐driven work investigating group differences in brain and behavior associations.

The current study split groups strictly on diagnostic outcome: infants who go on to have autism, infants who do not go on to have autism but have an autistic older sibling, and typically developing infants. Alternatively, a research domain criteria (RDoC) approach could be used in future work. RDoC was created by the National Institute of Mental Health to improve the research of mental disorders (Insel et al. 2010). Under RDoC—in an effort to fully capture the complexity of mental disorders—participants are characterized by five functional domains, rather than by strict diagnostic criteria (Cuthbert and Insel 2013). If an RDoC framework were applied to the current dataset, groups would not be based on positive or negative ASD diagnosis, but rather a dimensional approach would be used centered on language skills across all infants (Casey et al. 2014). Using an RDoC framework with data from the IBIS Network could potentially produce informative results and should be considered in future studies.

### 3.2 Strengths and Limitations

The study benefits from several strengths that enhance the reliability and validity of the findings. First, IBIS has taken great care to ensure uniformity and consistency in the data collection process. By employing identical scanners and sequences across all data collection sites, the study has minimized potential confounding factors related to different imaging protocols. Additionally, the use of geographic and traveling phantoms helps to standardize measurements and calibration, further improving the reliability of the data. All data were processed together, thus eliminating batch effects that might arise from processing data separately at different sites.

The sample size of the current project is large compared to many other similar infant neuroimaging studies. This larger number of participants increases the statistical power and generalizability of the results and therefore provides more robust conclusions about the relationships between morphometry and later language. However, recent evidence increasingly indicates that neuroimaging studies require even larger sample sizes (possibly in the thousands) to yield reproducible results (Marek et al. 2022). This limitation in sample size is closely related to the issue of data with (many) more variables than observations, an issue commonly called the “*P* >> *N*” or “large*‐P*, small*‐N*” problem—a situation that can lead to overfitting and reduced stability in traditional statistical models (Hastie et al. 2009). To address this limitation, we chose to employ PLSC because it is a dimension reduction method that can mitigate some of the issues caused by a large number of variables, including multicollinearity. The sample sizes of each group in this study are also highly unbalanced, so the power and generalizability of the results should not be considered the same for each group.

However, PLSC cannot directly model the longitudinal relationships between variables, so both time points were included in the analyses as separate variables. Future studies could use these results to conduct hypothesis‐driven analyses that directly investigate specific regions for longitudinal trajectories of morphometry and their relationship to language. Additionally, the 6 and 12 month morphometry measures used in this study are not directly comparable through items such as change scores because of the different processing steps that lead to their calculations. There is currently no statistical tool that allows for the analysis of longitudinal multi‐feature data with tests of group moderation.

The variables in the PLSC analyses were all residuals from linear regression analyses in order to control for several covariates. Therefore, the relationships captured by the latent variables and their loadings could be difficult to interpret when comparing to other studies that did not correct for the same covariates. Additionally, the loadings could be difficult to interpret, as they are interpreted using their magnitude and direction relative to each other (such as two high loadings with the same sign being positively associated) rather than with a numeric value (such as a correlation). There was also no formal testing to compare the diagnostic groups, as a separate PLSC analysis was run for each group. Nor were there tests of the specificity of the effects. Future efforts should aim to determine if other aspects of cognition are associated with cortical morphometry in the regions identified in this work.

A limitation that deserves recognition is the lack of diversity in our sample concerning race, ethnicity, and maternal education. Homogeneity in these demographic factors may restrict the generalizability of our findings to broader populations. To improve the external validity of future studies, it is important to include more diverse participants from various racial, ethnic, and socioeconomic backgrounds. Last, the “baby‐sibling” design has unique strengths and limitations. It is possible that the results of the current study only generalize to multiplex families, where there are two autistic children within the family unit. On the other hand, the baby‐sibling design is unbiased in terms of ascertainment because recruitment is not based on symptom presentation. Many community sample studies of autistic toddlers overrepresent those that have early appearing cognitive and language challenges, as they are more likely to be diagnosed with autism during toddlerhood.

## 4 Conclusion

Ultimately, the results of this study suggest that structures dedicated to language in infants who go on to have autism and in typically developing infants are not yet fully specialized. Given that the infant brain differs from the adult brain in notable ways, future studies investigating the biobehavioral correlates in infancy may benefit from using a global framework and not exclusively rely on adult models of neurobiology.

## Supporting information


**Table S1.** Significant regions that appear in more than one group.
**Table S2.** HL‐ASD Regions Comparison Mullen *T*‐scores vs. Mullen AE Scores.
**Table S3.** HL‐Neg Regions Comparison Mullen *T*‐scores vs. Mullen AE Scores.
**Table S4.** LL‐Neg Regions Comparison Mullen *T*‐scores vs. Mullen AE Scores.
**Figure S1.** Brain regions associated with language abilities, colored by their loadings on LV1, for all three groups. Blue regions were negatively associated, while red/orange regions were positively associated. The brain images are arranged into three columns and four rows. The columns are arranged by group with LL‐Neg on the left, HL‐Neg in the center, and HL‐ASD on the right. The rows are arranged by morphometry and time, with surface area at 6 months on the top row, surface area at 12 months on the second row, cortical thickness at 6 months on the third row, and cortical thickness at 12 months on the bottom row.

## Data Availability

The data that support the findings of this study are available from the corresponding author upon reasonable request.
