# Supplementary material for: Brain Morphometry in Infants Later Diagnosed With Autism is Related to Later Language Skills
Source: Hum Brain Mapp. 2025 May 9;46(7):e70221. doi: 10.1002/hbm.70221 (PMC12063482; doi:10.1002/hbm.70221)
Supplement: Supplementary file 1 — Table S1. Significant regions that appear in more than one group. Table S2. HL‐ASD Regions Comparison Mullen T‐scores vs. Mullen AE Scores. Table S3. HL‐Neg Regions Comparison Mullen T‐scores vs. Mullen AE Scores. Table S4. LL‐Neg Regions Comparison Mullen T‐scores vs. Mullen AE Scores. Figure S1. Brain regions associated with language abilities, colored by their loadings on LV1, for all three groups. Blue regions were negatively associated, while red/orange regions were positively associated. The brain images are arranged into three columns and four rows. The columns are arranged by group with LL‐Neg on the left, HL‐Neg in the center, and HL‐ASD on the right. The rows are arranged by morphometry and time, with surface area at 6 months on the top row, surface area at 12 months on the second row, cortical thickness at 6 months on the third row, and cortical thickness at 12 months on the bottom row. [file HBM-46-e70221-s001.docx]

SUPPLEMENT

*MRI Acquisition:*

Pediatric imaging was completed during natural sleep at each clinical site using identical 3-T Siemens TIM Trio scanners (Siemens Medical Solutions, Malvern, Pa.) equipped with 12-channel head coils. The imaging protocol included 1) a localizer scan, 2) 3D T1 MPRAGE: TR=2400ms, TE=3.16ms, 160 sagittal slices, FOV=256, voxel size = 1mm^3^, 3) 3D T2 FSE TR=3200ms, TE=499ms, 160 sagittal slices, FOV=256, voxel size = 1mm^3^, and 4) a 25 direction DTI: TR=12800ms, TE=102ms, slice thickness = 2mm isotropic, variable b value = maximum of 1000s/mm^2^, FOV=190.

*Image Preprocessing:*

All image processing was conducted blind to the participant group and diagnostic information. T1- and T2-weighted images were corrected for geometric distortions (Fonov et al. 2010) and intensity non-uniformity (Sled et al. 1998). T2-weighted images underwent linear, rigid registration to the corresponding T1-weighted images via mutual information registration. Subsequently, both T1- and T2-weighted images were transformed to stereotactic space based on the registration of the T1 scan. The skull was extracted using a “majority voting approach” between the T1 atlas mask, T2 atlas mask, and the T1 and T2 images jointly via FSL Brain Extraction Tool (Smith 2002). The resulting brain masks were manually corrected if necessary. All corrected and skull-stripped T1 and T2 images were used as input for an expectation, maximization-based, tissue segmentation tool (AutoSeg pipeline; https://www.nitrc.org/projects/autoseg/) to obtain white matter, gray matter, and CSF. See Kim et al. (2013) for more detail.

Cortical thickness and surface area measurements for each of the 78 brain regions were obtained via a CIVET workflow (Kim et al. 2005; MacDonald et al. 2000) adapted for this age using an age-corrected automated, anatomical labelling (AAL) atlas (Tzourio-Mazoyer et al. 2002). CIVET includes shrink-wrap deformable surface evolution of WM, local Laplacian distance and local surface area, mapping to spherical domain, co-registration using cortical sulcal features and extraction of regional measurements through a deformably co-registered fine-scale lobar parcellation. Surface area was measured at the mid-cortical surface. See Hazlett et al. (2017) for more detail.

*Supplemental analysis with Mullen age-equivalent scores*

One concern with using Mullen Expressive Language (EL) and Receptive Language (RL) *T*-scores are possible floor effects, especially in the HL-ASD group. In our sample of *n* = 31 HL-ASD participants, 6 were at floor level for the EL *T*-score and 14 were at floor level for the RL *T*-score. We investigated if using the Mullen age-equivalent (AE) scores, which do not contain floor effects in our sample, would notably impact the PLSC results obtained. We reran PLSC analyses for all three diagnostic groups using AE scores in place of *T*-scores.

The PLSC results for all three groups were very similar whether *T*-scores or AE scores were used. Loadings for LV1 language variables were very similar between the two types of analyses, with values that differed by an average of .014 (Table S5). The significant regions, based on the bootstrap ratios, differed slightly between the two types of analyses. Tables S2, S3, and S4 show the significant regions for each type of analysis, with an asterisk indicating that the loading was significant for that analysis. Across all regions, the loadings for LV1 were very similar between the two types of analyses, as each group’s analyses had a correlation of .99 between the loadings of the two types of analyses.

**Table S1.** Significant regions that appear in more than one group

| Region | Groups | Time | SA/CT | Loading |
| --- | --- | --- | --- | --- |
| Left anterior cingulate and paracingulate gyri | HL-Neg | 12 mo | CT | 0.111 |
|  | HL-ASD | 6 mo | SA | –0.105 |
| Right anterior cingulate and paracingulate gyri | LL-Neg | 12 mo | SA | –0.136 |
|  | HL-ASD | 6 mo | SA | –0.120 |
| Left fusiform gyrus | LL-Neg | 12 mo | SA | 0.106 |
|  | HL-ASD | 6 mo | CT | 0.148 |
|  | HL-ASD | 12 mo | SA | 0.121 |
|  | HL-ASD | 12 mo | CT | 0.094 |
| Left insula | LL-Neg | 6 mo | SA | 0.123 |
|  | HL-Neg | 12 mo | CT | 0.110 |
| Left inferior occipital gyrus | LL-Neg | 12 mo | CT | –0.099 |
|  | HL-ASD | 12 mo | SA | –0.100 |
| Left lingual gyrus | LL-Neg | 12 mo | CT | –0.141 |
|  | HL-Neg | 12 mo | SA | 0.098 |
| Left olfactory Cortex | HL-Neg | 12 mo | SA | –0.107 |
|  | HL-ASD | 6 mo | CT | 0.117 |
| Left superior frontal gyrus, medial orbital | HL-Neg | 12 mo | CT | 0.108 |
|  | HL-ASD | 6 mo | CT | 0.107 |
| Right posterior cingulate gyrus | HL-Neg | 12 mo | CT | –0.105 |
|  | HL-ASD | 12 mo | CT | –0.102 |
| Right parahippocampal gyrus | HL-Neg | 6 mo | SA | 0.113 |
|  | HL-Neg | 12 mo | SA | 0.130 |
|  | HL-ASD | 6 mo | SA | –0.122 |
|  | HL-ASD | 12 mo | SA | –0.143 |
| Right postcentral gyrus | LL-Neg | 6 mo | SA | –0.135 |
|  | HL-Neg | 12 mo | CT | 0.112 |
| Left precentral gyrus | LL-Neg | 6 mo | SA | –0.083 |
|  | HL-ASD | 6 mo | CT | 0.086 |
| Right Rolandic operculum | LL-Neg | 12 mo | SA | –0.116 |
|  | HL-Neg | 6 mo | CT | 0.113 |
|  | HL-Neg | 12 mo | CT | 0.097 |
|  | HL-ASD | 6 mo | SA | 0.082 |
| Right supplementary motor area | LL-Neg | 12 mo | SA | 0.109 |
|  | HL-Neg | 6 mo | CT | 0.102 |
| Right superior parietal gyrus | LL-Neg | 12 mo | SA | –0.115 |
|  | HL-ASD | 6 mo | CT | –0.080 |
|  | HL-ASD | 12 mo | CT | –0.108 |
| Right superior temporal gyrus | HL-Neg | 6 mo | SA | –0.120 |
|  | HL-ASD | 6 mo | CT | 0.098 |
| Right temporal pole: superior temporal gyrus | LL-Neg | 6 mo | CT | 0.080 |
|  | HL-Neg | 12 mo | SA | –0.112 |
|  | HL-ASD | 6 mo | SA | –0.096 |

**Table S2.** HL-ASD Regions Comparison Mullen *T*-scores vs Mullen AE Scores

| Time | Morphometry | Region | Loading *T*-score | Loading AE |
| --- | --- | --- | --- | --- |
| 6 mo | Surface Area | Left superior frontal gyrus, dorsolateral | -0.104* | -0.101* |
|  |  | Left inferior frontal gyrus, triangular part | -0.105* | -0.098* |
|  |  | Right Rolandic operculum | 0.082* | 0.077 |
|  |  | Left superior frontal gyrus, medial | -0.121* | -0.125* |
|  |  | Left anterior cingulate and paracingulate gyri | -0.105* | -0.098* |
|  |  | Right anterior cingulate and paracingulate gyri | -0.120* | -0.121* |
|  |  | Right parahippocampal gyrus | -0.122* | -0.123* |
|  |  | Left middle occipital gyrus | -0.087* | -0.099* |
|  |  | Left superior parietal gyrus | 0.077* | 0.076* |
|  |  | Right superior temporal gyrus | -0.060 | -0.068* |
|  |  | Right temporal pole: superior temporal gyrus | -0.096* | -0.082* |
|  |  | Right inferior temporal gyrus | 0.072 | 0.076* |
|  | Cortical Thickness | Left precentral gyrus | 0.086* | 0.078* |
|  |  | Left inferior frontal gyrus, triangular part | 0.104* | 0.107* |
|  |  | Left olfactory cortex | 0.117* | 0.114* |
|  |  | Left superior frontal gyrus, medial orbital | 0.107* | 0.108* |
|  |  | Right cuneus | -0.080* | -0.090* |
|  |  | Left fusiform gyrus | 0.148* | 0.142* |
|  |  | Right superior parietal gyrus | -0.080* | -0.081* |
|  |  | Right supramarginal gyrus | 0.100* | 0.095* |
|  |  | Right superior temporal gyrus | 0.098* | 0.085* |
|  |  | Left inferior temporal gyrus | 0.099* | 0.097* |
| 12 mo | Surface Area | Left precentral gyrus | -0.075 | -0.087* |
|  |  | Right precentral gyrus | 0.108* | 0.095* |
|  |  | Left inferior frontal gyrus, opercular part | 0.081 | 0.088* |
|  |  | Left parahippocampal gyrus | -0.128* | -0.131* |
|  |  | Right parahippocampal gyrus | -0.143* | -0.140* |
|  |  | Left middle occipital gyrus | -0.089* | -0.099* |
|  |  | Left inferior occipital gyrus | -0.100* | -0.092 |
|  |  | Left fusiform gyrus | 0.121* | 0.115* |
|  |  | Right superior parietal gyrus | 0.055 | 0.073* |
|  |  | Left precuneus | 0.077 | 0.088* |
|  |  | Left middle temporal gyrus | -0.084* | -0.073 |
|  | Cortical Thickness | Right posterior cingulate gyrus | -0.102* | -0.097* |
|  |  | Right cuneus | -0.091* | -0.094* |
|  |  | Right superior occipital gyrus | -0.120* | -0.128* |
|  |  | Right inferior occipital gyrus | -0.115* | -0.124* |
|  |  | Left fusiform gyrus | 0.094* | 0.072 |
|  |  | Right superior parietal gyrus | -0.108* | -0.101* |
|  |  | Right inferior parietal, but supramarginal and angular gyri | -0.089* | -0.087* |
|  |  | Right paracentral lobule | -0.079* | -0.104* |
|  |  | Left middle temporal gyrus | 0.110* | 0.118* |

**Table S3.** HL-Neg Regions Comparison Mullen *T*-scores vs Mullen AE Scores

| Time | Morphometry | Region | Loading *T*-score | Loading AE |
| --- | --- | --- | --- | --- |
| 6 mo | Surface Area | Left median cingulate and paracingulate gyri | -0.127* | -0.115* |
|  |  | Right median cingulate and paracingulate gyri | -0.110* | -0.099 |
|  |  | Left posterior cingulate gyrus | 0.103* | 0.087 |
|  |  | Right parahippocampal gyrus | 0.113* | 0.101* |
|  |  | Left inferior occipital gyrus | 0.080 | 0.086* |
|  |  | Right Heschl gyrus | -0.100* | -0.094* |
|  |  | Right superior temporal gyrus | -0.120* | -0.104* |
|  | Cortical Thickness | Right inferior frontal gyrus, orbital part | 0.105 | 0.104* |
|  |  | Right Rolandic operculum | 0.113* | 0.107* |
|  |  | Right supplementary motor area | 0.102* | 0.110* |
|  |  | Right insula | 0.135* | 0.144* |
|  |  | Right median cingulate and paracingulate gyri | 0.077 | 0.086* |
|  |  | Left cuneus | -0.112* | -0.116* |
| 12 mo | Surface Area | Right middle frontal gyrus | -0.105* | -0.115* |
|  |  | Left olfactory cortex | -0.107* | -0.106* |
|  |  | Right parahippocampal gyrus | 0.130* | 0.122* |
|  |  | Left lingual gyrus | 0.098* | 0.097* |
|  |  | Right temporal pole: superior temporal gyrus | -0.112* | -0.108* |
|  | Cortical Thickness | Left inferior frontal gyrus, orbital part | 0.120* | 0.116* |
|  |  | Right inferior frontal gyrus, orbital part | 0.090 | 0.095* |
|  |  | Left Rolandic operculum | 0.106* | 0.117* |
|  |  | Right Rolandic operculum | 0.097* | 0.099* |
|  |  | Left superior frontal gyrus, medial orbital | 0.108* | 0.103* |
|  |  | Left insula | 0.110* | 0.125* |
|  |  | Right insula | 0.105* | 0.115* |
|  |  | Left anterior cingulate and paracingulate gyri | 0.111* | 0.114* |
|  |  | Right posterior cingulate gyrus | -0.105* | -0.101* |
|  |  | Left postcentral gyrus | 0.106* | 0.107* |
|  |  | Right postcentral gyrus | 0.112* | 0.119* |
|  |  | Left superior parietal gyrus | 0.077 | 0.085* |

**Table S4.** LL-Neg Regions Comparison Mullen *T*-scores vs Mullen AE Scores

| Time | Morphometry | Region | Loading *T*-score | Loading AE |
| --- | --- | --- | --- | --- |
| 6 mo | Surface Area | Left precentral gyrus | -0.083* | -0.085* |
|  |  | Left insula | 0.123* | 0.137* |
|  |  | Right postcentral gyrus | -0.135* | -0.136* |
|  | Cortical Thickness | Right temporal pole: superior temporal gyrus | 0.080* | 0.074* |
| 12 mo | Surface Area | Right superior frontal gyrus, dorsolateral | -0.112* | -0.101 |
|  |  | Right Rolandic operculum | -0.116* | -0.114* |
|  |  | Right supplementary motor area | 0.109* | 0.108* |
|  |  | Right superior frontal gyrus, medial | -0.119* | -0.115* |
|  |  | Right anterior cingulate and paracingulate gyri | -0.136* | -0.136* |
|  |  | Right middle occipital gyrus | 0.142* | 0.137* |
|  |  | Left fusiform gyrus | 0.106* | 0.119* |
|  |  | Right superior parietal gyrus | -0.115* | -0.102 |
|  | Cortical Thickness | Left lingual gyrus | -0.141* | -0.148* |
|  |  | Left inferior occipital gyrus | -0.099* | -0.103* |
|  |  | Right fusiform gyrus | -0.180* | -0.185* |

**Table S5.** Language Loadings Comparison Mullen *T*-scores vs Mullen AE Scores

| Group | Language Measure | Loading *T*-score | Loading AE | Difference |
| --- | --- | --- | --- | --- |
| HL-ASD | Expressive | 0.700 | 0.698 | 0.002 |
|  | Receptive | 0.714 | 0.716 | 0.002 |
| HL-Neg | Expressive | 0.793 | 0.808 | 0.015 |
|  | Receptive | 0.609 | 0.589 | 0.020 |
| LL-Neg | Expressive | 0.733 | 0.754 | 0.021 |
|  | Receptive | 0.680 | 0.657 | 0.023 |

Figure S1 caption: Brain regions associated with language abilities, colored by their loadings on LV1, for all three groups. Blue regions were negatively associated, while red/orange regions were positively associated. The brain images are arranged into three columns and four rows. The columns are arranged by group with LL-Neg on the left, HL-Neg in the center, and HL-ASD on the right. The rows are arranged by morphometry and time, with surface area at 6 months on the top row, surface area at 12 months on the second row, cortical thickness at 6 months on the third row, and cortical thickness at 12 months on the bottom row.


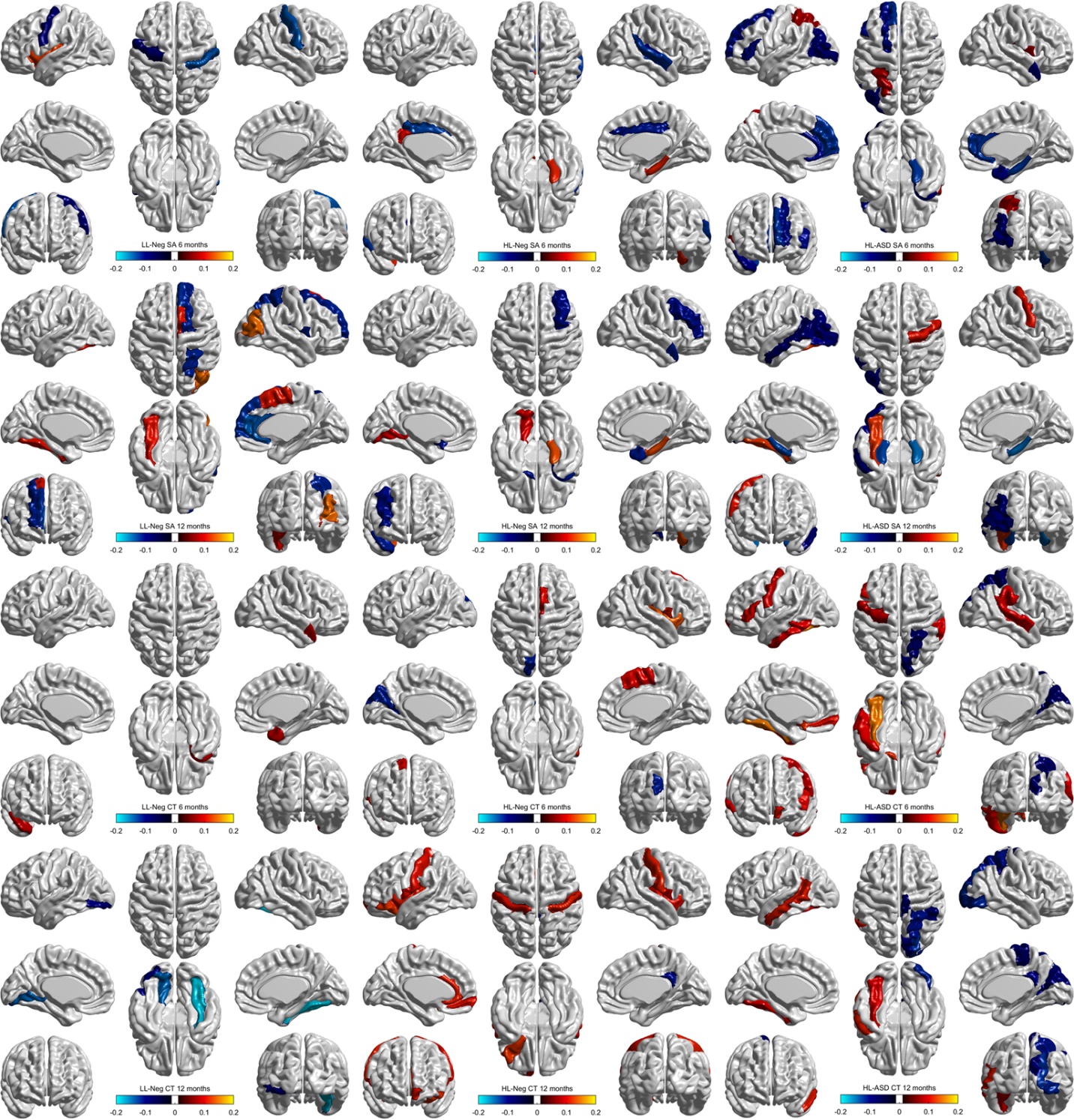


REFERENCES

Fonov VS, Janke A, Caramanos Z, Arnold DL, Narayanan S, Pike GB, Collins DL. 2010. Improved precision in the measurement of longitudinal global and regional volumetric changes via a novel MRI gradient distortion characterization and correction technique. In: Liao H, Edward PJ, Pan X, Fan Y, Yang GZ, editors. Medical Imaging and Augmented Reality. MIAR 2010. Lecture Notes in Computer Science. Springer Berlin Heidelberg. p.324-333

Hazlett HC, Gu H, Munsell BC, Kim SH, Styner M, Wolff JJ, Elison JT, Swanson MR, Zhu H, Botteron KN, et al. 2017. Early brain development in infants at high risk for autism spectrum disorder. Nature. 542(7641):348–351.

Kim JS, Singh V, Lee JK, Lerch J, Ad-Dab'bagh Y, MacDonald D, Lee JM, Kim SI, Evans AC. 2005. Automated 3-D extraction and evaluation of the inner and outer cortical surfaces using Laplacian map and partial volume effect classification. NeuroImage. 27(1):210–221.

Kim SH, Fonov VS, Dietrich C, Vachet C, Hazlett HC, Smith RG, Graves MM, Piven J, Gilmore JH, Dager SR, et al. 2013. Adaptive prior probability and spatial temporal intensity change estimation for segmentation of the one-year-old human brain. Journal of Neuroscience Methods. 212(1):43–55.

MacDonald D, Kabani N, Avis D, Evans AC. 2000. Automated 3-D extraction of inner and outer surfaces of cerebral cortex from MRI. NeuroImage. 12(1):340–356.

Sled JG, Zijdenbos AP, Evans AC. 1998. A nonparametric method for automatic correction of intensity nonuniformity in MRI data. IEEE Trans Med Imaging. 17(1):87–97.

Smith SM. 2002. Fast robust automated brain extraction. Human Brain Mapping. 17(3):143–155.

Tzourio-Mazoyer N, Landeau B, Papathanassiou D, Crivello F, Etard O, Delcroix N, Mazoyer B, Joliot M. 2002. Automated Anatomical Labeling of Activations in SPM Using a Macroscopic Anatomical Parcellation of the MNI MRI Single-Subject Brain. NeuroImage. 15(1):273–289.
